# Conservative treatment of knee arthrofibrosis according to a cellular cytokine-based model: A retrospective pilot evaluation

**DOI:** 10.1007/s00508-024-02497-0

**Published:** 2025-02-03

**Authors:** Robert Wakolbinger-Habel, Jakob Gaudernak, Brigitte Elisabeth Scheffold, Rainer Fiala, Robert Breuer, Martin Bittner-Frank, Clemens Lang, Helena Zehetner-Nics, Ana Oljaca, Mehdi Mousavi, Tatjana Paternostro-Sluga

**Affiliations:** 1 Clinic Donaustadt, Department of Physical and Rehabilitation Medicine (PRM), Vienna Healthcare Group, Langobardenstraße 122, 1220 Vienna, Austria; 2 Clinic Donaustadt, Department of Orthopaedics and Traumatology, Vienna Healthcare Group, Langobardenstraße 122, 1220 Vienna, Austria; 3 Clinic Floridsdorf, Department of Physical and Rehabilitation Medicine (PRM), Vienna Healthcare Group, Brünner Straße 68, 1210 Vienna, Austria; 4 External Lecturer, Medical University of Vienna, Währinger Gürtel 18-220, 1090 Vienna, Austria

**Keywords:** Arthrofibrosis, Stiffness, Joint mobility, Knee surgery, Total knee arthroplasty, Myofibroblasts

## Abstract

**Background:**

Arthrofibrosis (AF) is a relatively frequent complication after knee surgery, leading to painful limitation of joint mobility. Currently, intense stretching is performed in many patients, without improvements in mobility. A novel concept, established on a cellular, cytokine-based model, advocates to consider the warning function of pain and to terminate forced joint mobilization to disrupt the cycle of arthrofibrosis. Based on these findings, our clinic developed a comprehensive antifibrotic treatment protocol. The aim of this analysis was to evaluate the patients treated so far.

**Methods:**

Patients treated at the Clinic Donaustadt, an academic teaching hospital of the Medical University of Vienna, Austria, according to the clinic’s AF protocol, were evaluated by reviewing their records. Patellar mobility, range of motion, tightness, overheat, pain, general mobility, assistive devices, working status and the necessity of additional surgery were assessed at baseline, after 2 months and after 12 months (follow-up).

**Results:**

Four patients were treated according to the AF protocol. After two months, patellar mobility and range of motion clearly increased in three out of four patients. At the follow-up examination, patella mobility further increased, extension was complete, flexion at least 120°, and symptoms such as tightness, overheating, nocturnal pain and pain after activity had disappeared in three out of four patients. None of the patients had analgesics or walking aids and three out of four patients had returned to work. Out of the four patients, three expressed high satisfaction with the AF protocol and reported no limitations in daily life, including recreational activities.

**Conclusion:**

Based on this pilot evaluation, the comprehensive conservative AF program seems to have high potential. Therefore, larger future studies should be conducted to validate this concept.

## Background

Arthrofibrosis (AF) is one of the most frequent complications after knee surgery, such as anterior cruciate ligament repair or total knee arthroplasty (TKA). Concerning the epidemiology, the reported incidences range from 1–35% [1–6]; however, several authors also included patients with temporary stiffness after TKA. Therefore, the real incidence seems to be 3–4% [3].

Tightness as well as a painful limitation of joint mobility already develop in the early postoperative phase due to an increase in fibrous tissue [7].

Function is already impaired at early stages of AF: even in patients with a slight decrease of extension walking is affected and reduced flexion leads to difficulties in climbing stairs or sitting down. These limitations lead to impairments in instrumental activities of daily life, such as driving a car as well as in participation, including returning to work, with overall low quality of life [8].

Currently, the treatment is based on a pathophysiological adhesion model and usually involves forced stretching to regain the range of motion; however, intense classical physiotherapy including stretching or continuous passive motion is often painful and does not improve the range of motion but frequently even leads to a further decrease in function. In addition, manipulation under anesthesia and even surgical arthrolysis often only produce a short-term improvement in mobility and lead to unsuccessful treatment processes [9].

Relatively recent research advocates a new understanding of the so-called cellular cytokine-based pathogenesis of AF, which involves several proinflammatory cytokines, myofibroblasts and emotional distress [8, 9]. Therefore, several important aspects need to be acknowledged in the rehabilitation process, such as the warning function of pain, termination of forced joint mobilization, permitted motion within a pain-free range, balancing of the vegetative system and relaxation techniques [9, 10]. Currently, there is only little work in the literature on comprehensive conservative concepts for the treatment of AF patients.

Based on findings by Kieslich and Rückert [10] as well as on those by Traut [9], our clinic established a comprehensive antifibrotic treatment protocol to meet the special needs of patients with knee AF. The aim of this retrospective analysis was to evaluate the patients treated up to now.

## Methods

This retrospective study was conducted at the Clinic Donaustadt, an academic teaching hospital of the Medical University of Vienna, Austria. Before reviewing the records of the patients treated according to the clinic’s AF protocol, the approval by the local ethics committee was obtained. This research was performed in accordance with the ethical standards laid down in the 1964 Declaration of Helsinki and its later amendments.

Before diagnosing AF, other causes of knee stiffness had been ruled out, such as periprosthetic joint infection, implant instability, malpositioned or wrongly sized implants, neuroma, retropatellar arthrosis, complex regional pain syndrome and somatoform disorders. As there is no consensus on the clinical diagnosis of AF, the criteria were defined based on the recent literature [3, 9]. First, difficulty of pain control, particularly during mobilization; second, prolonged reduction in range of motion; third, feeling of tightness and fourth, an immobile patella.

Based on previous observations by Kieslich and Rückert [10], as well as by Traut [9] the protocol includes:

- patient education,
- cessation of painful stretching,
- soft gravity-based relaxation/stretching,
- passive patella mobilization,
- improvement of microcirculation by manual lymphatic drainages/connective tissue massages/painless electric stimulation (e.g., high tone therapy osteoarthritis program)/laser therapy/magnetic field therapy/reflexology/osteopathic soft tissue techniques,
- general relaxation techniques, autogenic training (with the aim of vagal nerve stimulation and sympathetic inhibition),
- medical training therapy (e.g., ergometer, resistance training), subaqua therapy, continuous passive motion (CPM; 30 min twice daily over 6 weeks at low velocity), gait training, mirror therapy (in cases of concomitant complex regional pain syndrome) strictly in a pain-free range and with no increase of feeling of tightness,
- no additional surgical procedure for at least 1 year.

The following parameters were investigated at baseline, after 2 months of treatment as well as at a follow-up examination after at least 12 months:

- patellar mobility (mediolateral and craniocaudal),
- range of motion,
- feeling of tightness,
- overheating,
- nocturnal pain, pain after activity, pain medication,
- general mobility, assistive devices such as crutches,
- working status.
- necessity for additional surgery.

In addition, overall satisfaction with the program was obtained via a 5-tier scale (1 = very unsatisfied, 5 = very satisfied).

## Statistics

Due to the small number of patients, data are presented descriptively. Range of motion was assessed via the neutral zero method, feeling of tightness via yes/no, clinical difference in skin temperature compared to the contralateral limb via yes/no. Mobility, necessity of aids and working status were described nominally. The necessity for operative revision was assessed via yes/no.

## Results

To date, four patients have been treated according to the AF protocol.

The baseline data are provided in Table 1.

**Table 1:** General medical data is presented for the four patients, as well as detailed information on baseline mobility and clinical symptoms

	Patient 1	Patient 2	Patient 3	Patient 4
Gender, age (years)	M, 68	F, 50	M, 53	F, 59
Cigarette smoking	–	–	20/day	–
Previous surgery	–	Anterior cruciate ligament repair (15 years ago)	Arthroscopic partial meniscectomy (3 years ago)	Mosaicplasty (14 years ago)
Chronic conditions	Atrial fibrillation	–	–	–
Surgery	Total knee arthroplasty; manipulation under anesthesia (6 weeks after surgery)	Total knee arthroplasty	Arthroscopic partial meniscectomy	Total knee arthroplasty; manipulation under anesthesia (8 weeks after surgery)
Time since surgery	2 months	1 month	2 months	3 months
Patella mobility medial-lateral (cm)	0.5	0.5	0	0
Patella mobility craniocaudal (cm)	0	0	0	0
Range of motion (degrees; neutral zero method)	0‑5-80	0‑15-85	0‑20-90	0‑15-90
Tightness	Yes	Yes	Yes	Yes
Overheating	Yes	Yes	Yes	Yes
Night pain	Yes	Yes	Yes	Yes
Pain after activity	Yes	Yes	Yes	Yes
Analgesics	No	Yes (metamizole, tramadol)	Yes (mefenamic acid)	Yes (naproxen, metamizole, tramadol)
Aids	Yes (crutches)	Yes (crutches)	Yes (crutches)	Yes (crutches)
Working status	Active	Sick leave	Sick leave	Sick leave

In two patients manipulation under anesthesia had already been performed, however, unsuccessfully. In patient 1 the range of motion before manipulation under anesthesia was 0‑10-65° and in patient 4 it was 0‑20-70°.

All patients had highly impaired craniocaudal patellar mobility and knee extension was incomplete. Moreover, all patients had a feeling of tightness, overheating compared to the contralateral knee, night pain as well as pain after activity.

After 2 months of treatment according to the AF concept, patella mobility increased in all patients: Mediolateral mobility doubled in two and tripled in one patient and craniocaudal mobility increased from 0 to 0.5 cm in three out of four patients.

The range of motion increased in most patients (15–35°), except for patient 4 where adherence to the program was low and she reported painful vigorous stretching exercises on a regular basis.

One patient reported the disappearance of tightness and overheating and two patients had no nocturnal pain anymore. One patient was pain-free after activity.

All patients discontinued both analgesics and walking aids.

One patient returned to work (Table 2).

**Table 2:** Clinical course after 2 months

	Patient 1	Patient 2	Patient 3	Patient 4
Patella mobility medial-lateral (cm)	1	1.5	1	0.5
Patella mobility craniocaudal (cm)	0.5	0.5	0.5	0
Range of motion (neutral zero method)	0‑0-110	0‑10-95	0‑15-100	0‑15-90
Improvement in extension (°)	5	5	5	0
Improvement in flexion (°)	30	10	10	0
Tightness	Yes	Yes	No	Yes
Overheating	Yes	Yes	No	Yes
Night pain	Yes	No	No	Yes
Pain after activity	No	Yes	Yes	Yes
Analgesics	No	No	No	No
Aids	No	No	No	No
Working status	Active	Sick	Sick	Sick

At the follow-up examination (Table 3) patella mobility further increased: Mediolateral mobility was 1.5–2.0 cm and craniocaudal 1.0–2.0 cm.

**Table 3:** Follow-up after 12 months

	Patient 1	Patient 2	Patient 3	Patient 4
Patella mobility medial-lateral (cm)	1.5	2.0	1.5	1.5
Patella mobility cranio-caudal (cm)	1.0	2.0	1.0	1.0
Range of motion (neutral zero method)	0‑0-120	0‑0-125	0‑0-140	0‑15-100
Improvement in extension (°) compared to baseline	5	15	20	0
Improvement in flexion (°) compared to baseline	40	40	50	10
Tightness	No	No	No	Yes
Overheating	No	No	No	Yes
Night pain	No	No	No	Yes
Pain after activity	No	No	No	No
Analgesics	No	No	No	No
Aids	No	No	No	No
Working status	Active	Active	Active	Sick
Additional surgery	No	No	No	Revision surgery (at another clinic)

Knee extension was complete in three out of four patients, and in most patients, flexion increased to at least 120°. In most patients, tightness, overheating, nocturnal pain and pain after activity had disappeared. None of the patients had analgesics or walking aids and three out of four patients had returned to work. Functionally, three out of four patients rated no limitations in daily life, including recreational activities. In particular, patient 1 returned to dancing on a regular basis. Patient 2 returned to recreational cycling and swimming. Patient 3 restarted long hiking tours.

In overall ratings, three out of four patients expressed high satisfaction (5 of 5 points) with the AF program. Patient 4 rated 3 of 5 points, i.e., neutral.

Concerning additional surgery, only patient 4 had an additional operation, i.e., revision surgery at another clinic. According to the other clinic’s discharge letter, the prosthesis had been too large and a CT-adjusted prosthesis was implanted. Nevertheless, neither range of motion nor the symptoms had clearly improved. The adherence to the program was still low and she continued painful vigorous stretching exercises on a regular basis.

## Discussion

This pilot evaluation demonstrated a high potential of the conservative AF concept. A clear improvement in most patients was already observed after 2 months, with further enhancement at the follow-up examination. Out of the four patients three were overall highly satisfied with the AF program and could resume recreational activities such as long hiking tours and dancing.

Patient 4 reported not only low adherence to the AF concept but also had a revision surgery at another clinic; however, thereafter neither the symptoms nor range of motion clearly improved. Therefore, we hypothesize that a significant part of her condition could be alleviated by adhering to our clinic’s AF protocol.

In order to facilitate a good prognosis it is essential to identify AF as early as possible. Even though the clinical diagnostic criteria of AF are not uniform in the literature some parameters are frequently mentioned [1, 3, 9]. First, pain control is difficult, particularly during mobilization. According to our observations there were high pain intensities even within the first postoperative week. Second, knee range of motion is decreased with a stronger decrease referring to more severe fibrosis. Third, the feeling of tightness is typical for AF. Fourth, an immobile patella is characteristic for AF.

According to an international consensus classification based on the extent of movement limitation, our patients had moderate AF [1]. In addition, potential biomarkers for AF after total knee arthroplasty, such as transforming growth factor-beta receptor 1, were immunohistochemically identified [5].

Before diagnosing AF, other causes of knee stiffness need to be ruled out, such as periprosthetic joint infection, implant instability, malpositioned or wrongly sized implants, neuroma, retropatellar arthrosis, complex regional pain syndrome, or somatoform disorders [3, 9].

Several invasive treatment options were reported, such as manipulation under anesthesia, arthroscopic or open adhesion lysis, as well as revision total knee arthroplasty [3]. Some orthopedic surgeons may use dexamethasone and ketorolac perioperatively when performing manipulation under anesthesia according to the Mayo Clinic protocol [2].

In two of our patients, manipulation under anesthesia had already been performed (without dexamethasone or ketorolac). Some patients may benefit from manipulation under anesthesia. According to a recent review [3], the average gains in range of motion are 22–39° for flexion and 2–5° for extension, with failure rates of 7–24%. Even though on a low level potential complications, such as fractures, wound dehiscence, patellar tendon avulsion, and hemarthrosis need to be considered.

In addition, other authors reported a high relapse rate after manipulation under anesthesia [9] and in both of our patients, the procedure was unsuccessful.

In contrast, the conservative AF program was effective: In most patients, clear gains of 10–30° for flexion and 5° for extension were already observed after 2 months, with further increases to 40–50° for flexion and 5–20° for extension after 12 months.

Therefore, the cellular cytokine-based model of pathophysiology needs to be kept in mind. According to this model, mechanical stress (such as vigorous stretching exercises or manipulation under anesthesia) can maintain the activation of myofibroblasts and thereby AF. This model can explain the unsuccessful course in some patients after manipulation under anesthesia and in these patients our clinic’s concept may be a reasonable alternative.

There is relatively little work on comprehensive conservative treatment concepts [9, 10]. Some authors advocate aggressive physiotherapy [3]; however, aggressive approaches may again trigger myofibroblasts due to high mechanical stress. The debate on CPM is controversial [3]. In our patients, CPM was performed for 30 min twice daily over 6 weeks carefully and at low velocity without provoking pain or tightness.

Pharmacological interventions, such as prednisolone and propranolol are off-label symptomatic treatments without directly targeting myofibroblasts. Therefore, they are not classified as causal treatment options and their effect seems to be lower compared to correct behavior strategies and physiotherapy [9]. Therefore, drugs were not included in this AF concept at our clinic.

Based on previous observations [9, 10], we established a comprehensive AF program at our clinic. According to this protocol the first important step is patient education on AF causes and treatment options. This is the basis for achieving correct behavior strategies, such as the cessation of painful stretching. Moreover, soft gravity-based relaxation/stretching techniques are implemented as well as passive patella mobilization. These techniques must not cause any pain, otherwise AF could be maintained.

In addition, microcirculation should be improved, e.g., by manual lymphatic drainages, laser therapy, magnetic field therapy, osteopathic soft tissue techniques or painless electric stimulation (e.g., via the osteoarthritis program of high tone therapy). High tone therapy, a specific type of electrotherapy, delivers medium frequency alternating current. The carrier frequency ranges from 4 kHz to 33 kHz and both frequency and amplitude are modulated simultaneously (simulFAM ®; HiToP ® 4 touch, GBO Medizintechnik AG, Rimberg, Germany). In the so-called “osteoarthritis program”, both low and medium frequency currents are combined, thereby using both the low frequency gate control concept of analgesia, as well as the regenerative effects of the simulFAM i ® medium frequency current, as special form of simulFAM ® [11].

Furthermore, the vegetative nervous system should be balanced, e.g., by general relaxation techniques, reflexology, connective tissue massages or autogenic training (with the aim of vagal nerve stimulation and sympathetic inhibition).

Finally, medical training therapy is an important part of the program. Ergometer training and resistance training should be performed as well as subaqua therapy and gait training. Continuous passive motion (CPM) was part of the program as well and in cases of concomitant complex regional pain syndrome, also mirror therapy should be implemented. Again, it is emphasized that the training needs to be strictly kept in a pain-free range and the feeling of tightness must not increase. Finally, there should be no additional surgical procedure for at least 1 year.

In summary, the findings of this pilot evaluation are promising. Most patients were highly satisfied and clear improvements of the symptoms as well range of motion were observed. Therefore, this comprehensive AF program could serve as an alternative for those patients who fail to benefit from manipulation under anesthesia. Moreover, our concept could help to prevent the development of AF when implemented early in the course of the disease.

Therefore, our clinic’s concept should be evaluated in larger studies to confirm the positive effects. In particular, differences between patients with and without previous manipulation under anesthesia and with and without risk factors such as cigarette smoking and diabetes should be addressed as well as dose-response relationships of the different treatments, such as electrotherapy and continuous passive motion. Additionally, potential additional benefits by specific medications such as prednisolone and propranolol should be investigated.

## References

[1] Kalson NS, Borthwick LA, Mann DA, Deehan DJ, Lewis P, Mann C, et al. International consensus on the definition and classification of fibrosis of the knee joint. Bone Joint J. 2016;98-B:1479–88. 27803223 10.1302/0301-620X.98B10.37957

[2] Lee DR, Therrien E, Song BM, Camp CL, Krych AJ, Stuart MJ, et al. Arthrofibrosis nightmares: prevention and management strategies. Sports Med Arthrosc Rev. 2022; 10.1097/JSA.0000000000000324. 35113841 10.1097/JSA.0000000000000324 PMC8830598

[3] Pasqualini I, Surace PA, Molloy RM, Deren ME, Piuzzi NS. Arthrofibrosis after total knee arthroplasty: a critical analysis review. JBJS Rev. 2023; 10.2106/JBJS.RVW.23.00140. 38079496 10.2106/JBJS.RVW.23.00140

[4] Salmons HI, Payne AN, Taunton MJ, Owen AR, Fruth KM, Berry DJ, et al. Nonsteroidal anti-inflammatory drugs and oral corticosteroids mitigated the risk of arthrofibrosis after total knee arthroplasty. J Arthroplast. 2023;38:S350–S4. 10.1016/j.arth.2023.03.076 PMC10430696 37011702

[5] Chen X, Wang Z, Huang Y, Deng W, Zhou Y, Chu M. Identification of novel biomarkers for arthrofibrosis after total knee arthroplasty in animal models and clinical patients. eBioMedicine. 2021;70:103486. 34311327 10.1016/j.ebiom.2021.103486 PMC8325099

[6] Schneider AM, Rice SJ, Lancaster N, McGraw M, Farid Y, Finn HA. Low-dose irradiation and rotating-hinge revision for the treatment of severe idiopathic arthrofibrosis following total knee arthroplasty: a review of 60 patients with a mean 6‑year follow-up. J Arthroplast. 2024;39:1075–82. 10.1016/j.arth.2023.10.021 37863275

[7] Gollwitzer H, Burgkart R, Diehl P, Gradinger R, Bühren V. Therapie der Arthrofibrose nach Kniegelenkendoprothetik. Orthopade. 2006;35:143–52. 10.1007/s00132-005-0915-5. 16374640 10.1007/s00132-005-0915-5

[8] Usher KM, Zhu S, Mavropalias G, Carrino JA, Zhao J, Xu J. Pathological mechanisms and therapeutic outlooks for arthrofibrosis. Bone Res. 2019; 10.1038/s41413-019-0047-x. 30937213 10.1038/s41413-019-0047-x PMC6433953

[9] Traut P. Klinische Diagnostik, Differenzialdiagnostik, Pathogenese- und Stadienmodell der Arthrofibrose. Unfallchirurgie. 2022;125:839–48. 10.1007/s00113-022-01237-1. 36107205 10.1007/s00113-022-01237-1 PMC9633511

[10] Kieslich K, Rückert U. Prevention, physiotherapeutic preoperative and postoperative treatment and rehabilitation of arthrofibrosis. Unfallchirurgie. 2022;125:849–55. 10.1007/s00113-022-01247-z. 36197504 10.1007/s00113-022-01247-z

[11] Wilk M, et al. The application of high-tone power therapy in the rehabilitation of patients with soft-tissue damage to the knee joint. Fizjoterapia Polska. 2002;2(2):118.

